# Genetic variation in the genome-wide predicted estrogen response element-related sequences is associated with breast cancer development

**DOI:** 10.1186/bcr2821

**Published:** 2011-01-31

**Authors:** Jyh-Cherng Yu, Chia-Ni Hsiung, Huan-Ming Hsu, Bo-Ying Bao, Shou-Tung Chen, Giu-Cheng Hsu, Wen-Cheng Chou, Ling-Yueh Hu, Shian-Ling Ding, Chun-Wen Cheng, Pei-Ei Wu, Chen-Yang Shen

**Affiliations:** 1 Department of Surgery, Tri-Service General Hospital, Ming-Cheng East Road, Taipei, 114, Taiwan; 2 Institute of Biomedical Sciences, Academia Sinica, Academy Street, Taipei, 115, Taiwan; 3 Department of Pharmacy, China Medical University, Hsueh-Shih Road, Taichung, 404, Taiwan; 4 Department of Surgery, Changhua Christian Hospital, Nanxiao Street, Changhua, 500, Taiwan; 5 Department of Radiology, Tri-Service General Hospital, Ming-Cheng East Road, Taipei, 114, Taiwan; 6 Department of Nursing, Kang-Ning Junior College of Medical Care and Management, Kangning Road, Taipei, 114, Taiwan; 7 Institute of Biochemistry and Biotechnology, Chung Shan Medical University, Jianguo North Road, Taichung, 402, Taiwan; 8 Graduate Institute of Environmental Sciences, China Medical University, Hsueh-Shih Road, Taichung, 404, Taiwan

## Abstract

**Introduction:**

Estrogen forms a complex with the estrogen receptor (ER) that binds to estrogen response elements (EREs) in the promoter region of estrogen-responsive genes, regulates their transcription, and consequently mediates physiological or tumorigenic effects. Thus, sequence variants in EREs have the potential to affect the estrogen-ER-ERE interaction. In this study, we examined the hypothesis that genetic variations of EREs are associated with breast cancer development.

**Methods:**

This case-control study involved 815 patients of Asian descent with incident breast cancer and 821 healthy female controls. A total of 13,737 ERE sites in the whole genome predicted by a genome-wide computational algorithm were blasted with single-nucleotide polymorphism (SNP) sequences. Twenty-one SNPs located within 2,000 bp upstream or within introns 1 and 2 of putative genes and with a minor allele frequency greater than 5% were identified and genotyped. Frequencies of SNPs were compared between cases and controls to identify SNPs associated with cancer susceptibility.

**Results:**

A significant combined effect of rs12539530, an ERE SNP in intron 2 of *NRCAM *which codes for a cell adhesion molecule, and SNPs of *ESR1*, the gene coding for ER, on breast cancer risk was found. Interestingly, this combined effect was more significant in women who had experienced a longer period of lifetime estrogen exposure, supporting a hormonal etiology of this SNP in breast tumorigenesis.

**Conclusions:**

Our findings provide support for a role of genetic variation in ERE-*ESR1 *in determining susceptibility of breast cancer development.

## Introduction

The roles of estrogen receptor α (ERα) in initiating tumor development in breast cancer, regulating progression and determining therapeutic protocols and efficacy are well documented [1-3]. Although ERα can be activated in an estrogen-independent manner, the classical activation mechanism involves ERα binding to estrogen and other coactivator proteins to form the estrogen-bound ER complex, which functions as a transcriptional regulator [4,5]. The DNA-binding domain of ERα binds to estrogen response elements (EREs) in the promoter region of estrogen-responsive genes, activating or repressing their transcription and consequently mediating physiological or tumorigenic effects. Given that sequence variants, such as single-nucleotide polymorphisms (SNPs), located in the promoters of genes have the potential to affect the protein (transcription factor)-DNA (promoter) interaction, resulting in altered expression of target genes [6,7], we decided that it was meaningful to examine the hypothesis that genetic variations of EREs might be associated with breast cancer development.

Early work on the *Xenopus *vitellogenin gene identified a minimal ERE core sequence of 5'-GGTCANNNTGACC-3' [8]. Since then, several computational approaches have been used to map EREs on a genome-wide level on the basis of the presence of EREs within promoter proximal regions [9,10]. By specifically focusing on promoter regions, 12,515 EREs have been identified in the human genome [10-12]. To distinguish between real binding sites and noise, several attempts have been made to improve the specificity of prediction. For instance, by eliminating EREs that are not conserved between the human and mouse genomes, the number of gene proximal EREs has been reduced to 660 [10,11]. In this study, we used PReMod [13], a new database of genome-wide *cis*-regulatory modules, to predict all possible EREs in the genome. The prediction algorithm of PReMod takes into account the fact that, in higher eukaryotes, *cis*-regulatory regions often contain several phylogenetically conserved binding sites for different transcription factors [13,14], and thus it has proven to be more reliable than other methods. Using the SNPs of the human genome available in databases, we searched for SNPs within these genome-wide predicted EREs and explored their association with breast cancer.

## Materials and methods

### Study participants

This case-control study is part of an ongoing cooperative study aimed at understanding the causes of breast cancer in Taiwan. Breast cancer in Taiwan is characterized by low incidence, early tumor onset, hormone dependency and novel genomic alterations [15-17]. We studied 815 female breast cancer patients with pathologically confirmed incident primary breast cancer seen at the Tri-Service General Hospital or the Changhua Christian Hospital between March 2002 and August 2007. The 821 healthy female controls were selected from among women attending the health examination clinics of the same hospitals during the same period. The characteristics of these study participants have already been described in detail [18-21], and some participants (551 patients and 727 controls) have recently been genotyped for polymorphism of *ESR1 *[21], the gene coding for the ER. This study was approved by the Ethics Committee of the Institutional Review Board of the Academia Sinica, Taiwan, and informed consent was obtained from all study participants before the collection of epidemiologic data by personal interview. Considerations regarding methodological issues in the present study (such as study design, sampling scheme, and potential bias) have been described in detail previously [18-21].

### Questionnaire

Experienced research nurses were assigned to administer a structured questionnaire to both patients and controls. The information collected has been described, and the validity of the questionnaire has been addressed and confirmed, in our previous studies [18-21].

### Specimen collection and SNP selection and genotyping

At the end of the interview, blood was taken for DNA isolation and genotyping. All samples were examined by laboratory personnel who were blinded to the case/control status of specimens. DNA was extracted from the peripheral blood samples of patients and controls using DNA purification kits (Promega, Madison, WI, USA).

The PReMod database is a genome-wide/transcription factor-wide collection of more than 100,000 computational predicted transcriptional regulatory modules within the human genome [13,14]. These modules are specific sequences potentially regulated by 229 transcription factor families, and the PReMod algorithm predicts that a total of 13,737 sites within the human genome are bound and/or regulated by the ER [13,14]. We compared these sites with conventional SNP databases [22-24] and identified ER-binding sites potentially harboring SNPs. The following three criteria were then used to determine the SNPs to be genotyped: (1) because of statistical considerations (that is, consideration of study power), the minor allele frequency of the selected SNPs had to be higher than 0.05; (2) because of biological considerations, the selected SNPs had to be located within 2,000 bp upstream or located within introns 1 and 2 of putative genes; and (3) because of technical considerations, SNPs having the potential to yield a false signal using the iPLEX high-throughput genotyping platform were excluded. As a result, a total of 21 SNPs were chosen for genotyping.

SNPs were genotyped in all samples using Sequenom iPLEX technology (Sequenom, Hamburg, Germany). Positive, negative and duplicate controls were included on all plates, with genotypes being autocalled by specialized software (MassARRAY Typer version 3.4; Sequenom) and subsequently confirmed by visual assessment of the data. All assays were performed by individuals blinded to the case-control status of the samples. As a quality control, we repeated the genotyping on 10% of the samples, and all genotype scoring was performed and checked separately by one reviewer who was unaware of the case-control status. The concordance rate for replicate samples was 100%.

### Statistical analysis

Univariate and multivariate analyses were used to determine the risk factors for breast cancer in this series of study participants, and the odds ratio (OR) and corresponding 95% confidence intervals (95% CIs) were estimated. For individual ERE SNPs, genotype frequencies were assessed for departure from the Hardy-Weinberg equilibrium using either a χ^2 ^goodness-of-fit test or a Fisher's exact test. The χ^2 ^test for a 2 × 3 contingency table was used to compare genotype frequency between patients and controls. To take account of multiple comparisons, these associations were also assessed using the permutation test provided in the Haploview software tool (Broad Institute of the Massachusetts Institute of Technology and Harvard University, Cambridge, MA, USA), run using 10,000 permutations. The association of susceptibility genotypes and breast cancer risk was further evaluated with simultaneous consideration of established risk factors for breast cancer or other significant risk factors in multivariate logistic regression models. Biologic plausibility was the most important criterion for inclusion of variables in the model. Therefore, we included all established risk factors for breast cancer in the statistical models: age, family history of breast cancer, age at menarche, parity and age at first full-term pregnancy (FFTP). Adjusted ORs (aORs) and 95% CIs for genotypes were then estimated.

We made use of the information on the *ESR1 *polymorphism in our study participants that we published recently [21] and explored the effect of a possible *ESR1*-ERE interaction or estrogen-*ESR1*-ERE interaction in determining breast cancer development. A combination of the joint method and stratified analysis [18-21] was applied to determine whether this interaction between *ESR1 *and ERE was associated with breast cancer formation. A joint effect of *ESR1*-ERE on increased breast cancer risk was explored using conventional logistic regression, a test evaluating whether a statistically significant increase in risk was observed with specific combinations of putative high-risk genotypes in these SNPs (measured by the β estimates from this regression model). In addition, we stratified our study participants on the basis of *ESR1 *genotype and examined whether breast cancer risk associated with ERE SNPs was particularly significant in specific *ESR1 *genotype subsets of women. Because we were especially interested in the relationship between the joint effect of *ESR1*-ERE and breast cancer risk within categories of risk factors representing different levels of estrogen exposure, we performed stratified analysis to test this hypothesis. Therefore, if the identified joint effect of *ESR1*-ERE SNPs initiated breast cancer by the formation of the estrogen-ER complex, then the relationship between breast cancer risk and the joint effect would not be the same in women who had experienced different lengths of estrogen exposure. This was evaluated by calculating the risk (aOR) of breast cancer associated with the joint effect of *ESR1*-ERE SNPs in women with a longer or shorter period of total estrogen exposure. For menopausal women, total estrogen exposure was calculated using the formula (age at menopause - age at menarche - years of full-term pregnancy) and, for premenopausal women, age at menopause in this formula was replaced by age at recruitment into this study.

## Results

The risk profile of breast cancer in our study participants was similar to that found in our previous studies [18-21] and in other breast cancer studies. The development of breast cancer was found to be highly associated with reproductive risk factors, including early menarche, nulliparity, lower number of full-term pregnancies (FTPs) and older age at FFTP (Table 1). Compared to controls, patients were younger at menarche (≤ 14 years vs. >14 years, aOR, 1.52; 95% CI, 1.18 to 1.96) and older at FFTP (>23 years vs. ≤ 23 years, aOR, 1.30; 95% CI, 1.00 to 1.69). Significant protection was conferred by a history of FTP (parous women vs. nulliparous women, aOR, 0.67; 95% CI, 0.46 to 0.97) and a greater number of FTPs (history of more than two FTPs vs. history of two or fewer FTPs, aOR, 0.46, 95% CI, 0.34 to 0.61). No association was found between cancer risk and a history of oral contraceptive use or between cancer risk and body mass index. Significant risk factors were included in the multivariate logistic regression models when we examined the association between SNPs and cancer risk. More importantly, these significant associations between reproductive risk factors and breast cancer reveal the importance of the estrogen-related etiology of breast cancer in our participants, providing the opportunity to examine the contribution of EREs during breast tumorigenesis.

**Table 1:** Frequency of risk factors in breast cancer patients and controls and the adjusted odds ratios in relation to breast cancer risk^a^

Risk factor	Patients, % (*n *= 814)	Controls, % (*n *= 821)	**aOR (95% CI)**^**b**^
Age at menarche			
>14 yr	34.1	37.6	1.00 (reference)
≤ 14 yr	65.9	62.4	1.52 (1.18 to 1.96)
Menopause			
No	49.6	59.5	1.00 (reference)
Yes	50.4	40.5	0.60 (0.42 to 0.84)
Family history of breast cancer in first-degree relatives			
No	88.7	90.7	1.00 (reference)
Yes	11.3	9.3	1.20 (0.83 to 1.73)
Nulliparity			
No	11.6	10.2	1.00 (reference)
Yes	88.4	89.8	0.67 (0.46 to 0.97)
No. of full-term pregnancies			
≤ 2	51.0	45.6	1.00 (reference)
>2	49.0	54.4	0.46 (0.34 to 0.61)
Age at first full-term pregnancy			
≤ 23 years	62.6	64.0	1.00 (reference)
>23 years	37.4	36.0	1.30 (1.00 to 1.69)
Body mass index			
≤ 23	56.9	59.1	1.00 (reference)
>23	43.1	40.9	0.90 (0.71 to 1.15)
Use of oral contraceptives			
No	81.4	82.1	1.00 (reference)
Ever	18.6	17.9	1.05 (0.78 to 1.40)

^a^aOR, adjusted odds ratio; 95% CI, 95% confidence interval; ^b^aOR and 95% CI were estimated in a logistic regression model in which the age of study participants was included.

**Figure 1:**
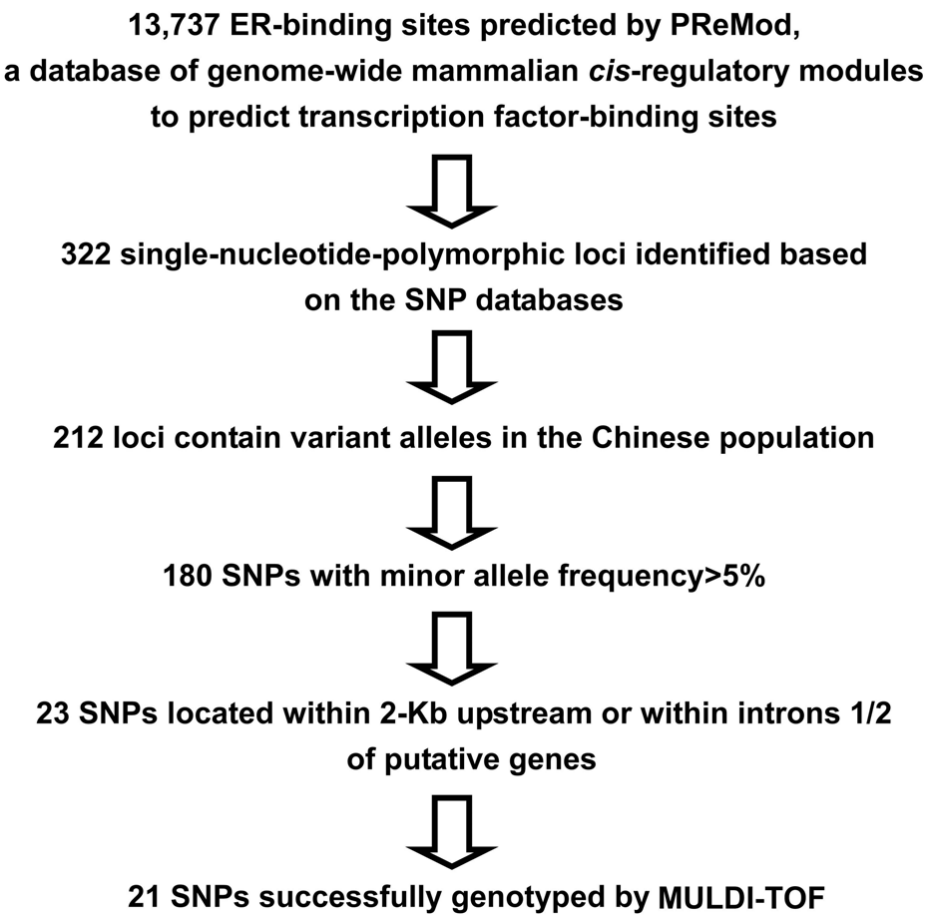
**Flow diagram showing selection from the genome-wide-predicted estrogen response element-related sequences of single-nucleotide polymorphisms (SNPs) for genotyping**. ER, estrogen receptor; MULDI-TOF, matrix-assisted laser desorption inoization-time of flight mass spectrometry.

A total of 13,737 sites (274,740 bp of DNA) in the whole genome were predicted to be ERE-related sequences using the PReMod algorithm. After blasting these data with online information available from SNP data sets (University of California, Santa Cruz, National Center for Biotechnology Information and HapMap), 322 ER-binding sites were identified as potentially harboring SNPs. One hundred ten of these 322 SNPs were found to contain no variant allele in the Chinese population, resulting in 212 SNPs, 21 of which met our criteria and were genotyped in patients and controls (Figure 1).

**Figure 2:**
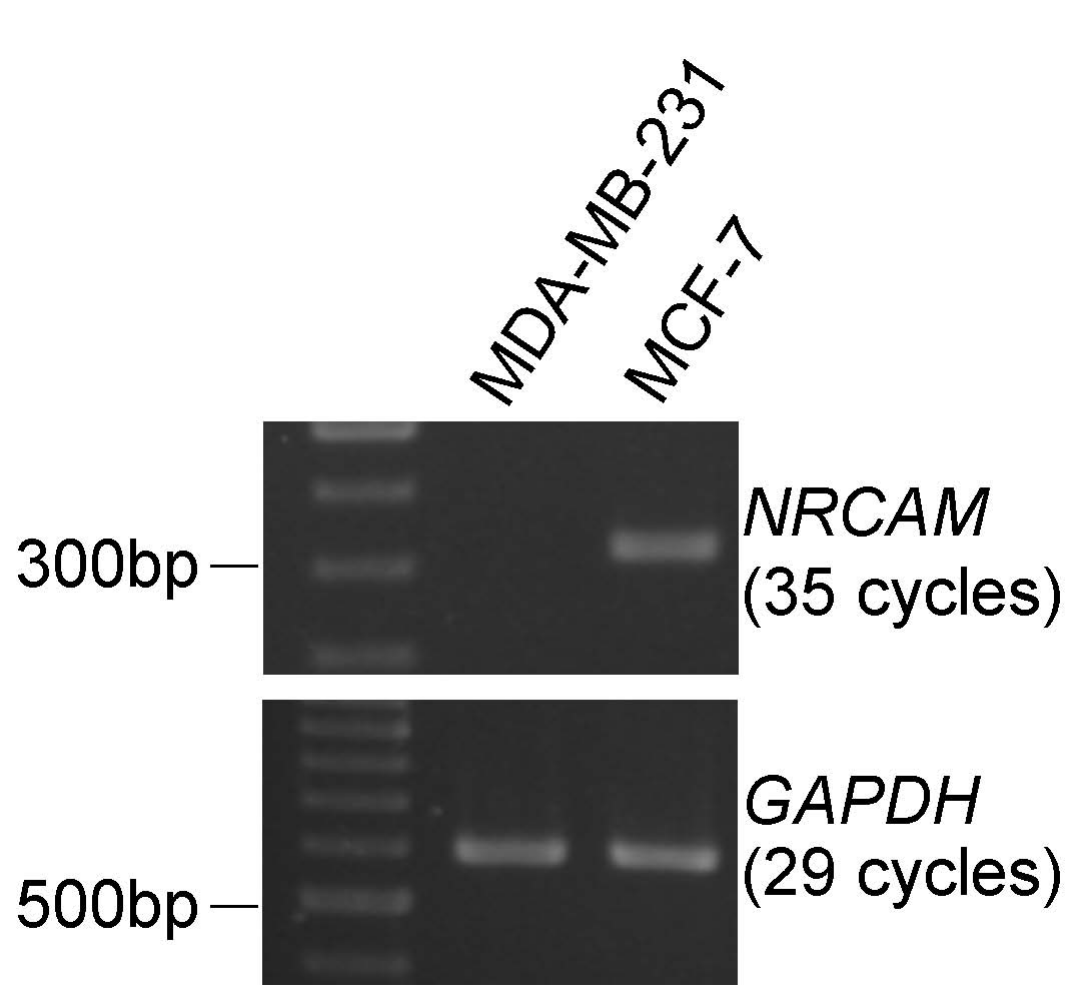
**Transcription (mRNA) of *NRCAM *detected by reverse transcriptase-polymerase chain reaction (RT-PCR) assay is seen in the estrogen receptor (ER)-positive breast cancer cell line MCF-7, but not in the ER-negative breast cancer cell line MDA-MB-231**. *GAPDH *is the positive (mRNA) control of RT-PCR. For RT-PCR, total RNA was extracted from cell lines using the Arcturus PicoPur RNA Isolation Kit (Applied Biosystems, Forster City, CA, USA), and to convert RNA into cDNA, reverse transcription was performed for 70 minutes at 42°C in a reaction volume of 20 μl containing 1 μg of RNA, 10 mM random oligo(dT) primer (Promega), and 5 U of SuperScript II reverse transcriptase (Gibco-BRL, Gaithersburg, MD, USA). The presence of a cDNA band of the appropriate molecular weight was determined on 1% agarose gel after electrophoresis.

To determine the breast tumorigenic contribution of ERE SNPs, we examined whether the genotypic distribution of individual SNPs differed between the cases and controls (Table S1 in Additional file 1). The frequencies of all SNPs in the controls agreed with those expected on the basis of the Hardy-Weinberg equilibrium, suggesting that genotyping error was relatively unlikely. The result for the genotypic analysis of two SNPs (rs12539530 and rs9527676) was important, as it showed that women carrying the homozygous variant genotype had an significantly increased OR (*P *< 0.05) compared with women carrying the homozygous wild-type genotype, as well as that the carrying of one additional risk allele was associated with a significant increase in risk (Table S1 in Additional file 1). The possibility of false-positives due to multiple testing is less likely, because the permutation test showed on the basis of 10,000 random permutations that these two associations were borderline significant or significant (*P *= 0.08 and *P *= 0.03, respectively) (Table S1 in Additional file 1). Both SNPs are located in regulatory regions of genes coding for cell adhesion molecules, as rs12539530 is located in intron 2 of *NRCAM*, a gene coding for a neuron-related cell adhesion molecule, and rs9527676 is located in intron 1 of *PCDH17*, a gene coding for protocadherin-17. To gain initial clues for further analysis, we examined the expression of these two genes in breast cancer cell lines. To this end, we checked the expression of *NRCAM *and *PCDH17 *in breast cancer cell lines expressing *ESR1 *(MCF-7 cells) or not expressing *ESR1 *(MDA-MB-231 cells) and examined whether the expression of these putative ER-regulated genes is ER-dependent. The results for *NRCAM *were more promising, as they showed that this gene could be expressed in an ER-positive breast cancer cell (Figure 2), which is consistent with previous findings using microarray technology [25,26]. In addition, cell-cell adhesion, in which *NRCAM *is known to play a part, has been well documented as being involved in cancer formation [27,28]. All of these lines of evidence support the biological plausibility of our findings, suggesting that rs12539530, found in an ERE-related sequence and possibly regulating *NRCAM *expression, is associated with breast cancer susceptibility.

**Figure 3:**
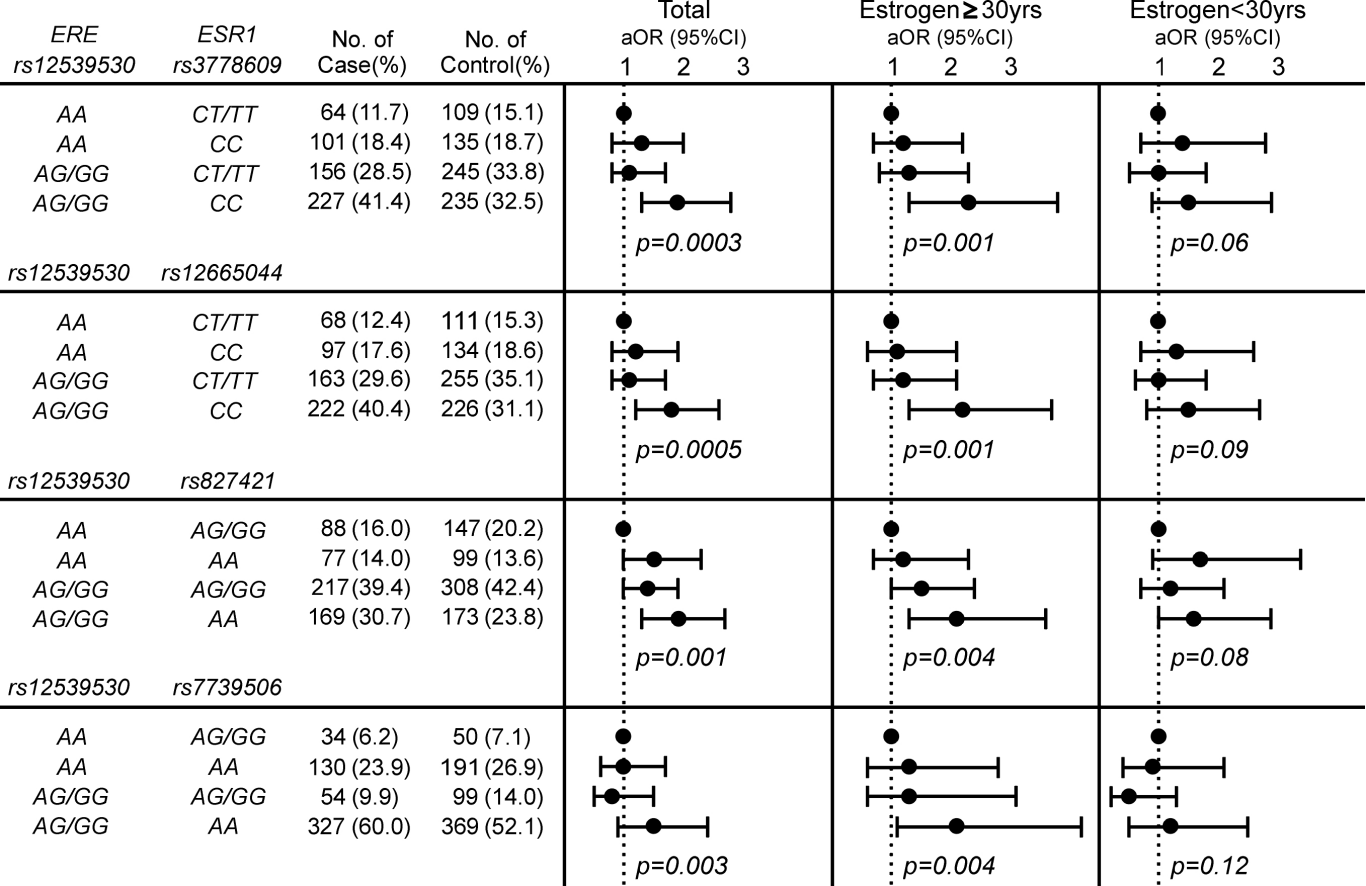
**Association of the joint effect of genotypes of rs12539530, a single-nucleotide polymorphism (SNP) associated with estrogen response elements (EREs), and of single-nucleotide polymorphisms of *ESR1*, the estrogen receptor gene, with breast cancer risk in all women combined and in women stratified by total years of estrogen exposure (indicated as "Estrogen")**. aOR, adjusted odds ratio; 95% CI, 95% confidence interval.

The well-known mechanism in which ER binds to EREs to mediate the expression of ER-regulated genes [2-4] prompted us to speculate whether the SNPs of EREs and *ESR1 *might be jointly associated with breast cancer. We made use of the information on the *ESR1 *genotype of our study participants that we published recently [21] and examined this possibility using both stratified analysis and the joint method. Three SNPs (rs3778609, rs12665044, and rs827421) located in one cluster in intron 1 within the sequence coding for the activation function (AF)-1 domain of the ER and one SNP (rs7739506) located in intron 4 within the sequence coding for the AF-2/ligand-binding domain have previously been found to show significant or borderline significant associations with breast cancer susceptibility in our population [21]. If this ERE SNP were linked to breast cancer susceptibility via the suspected ER-related mechanism, the association between rs12539530 and breast cancer would differ between women harboring different *ESR1 *genotypes. Our findings are consistent with this speculation, and the association between high-risk genotypes of rs12539530 and an increased breast cancer risk was significant in only one subset of women carrying specific genotypes of *ESR1*, but not in the other subset (Table S2 in Additional file 1). Furthermore, on the basis of a very stringent multiplicative model, the borderline significance *P *values for the interaction between rs12539530 and rs827421 (*P *= 0.07) and between rs12539530 and rs7739506 (*P *= 0.09) are in line with the suggestion that an interaction between rs12539530 and *ESR1 *polymorphism is linked to breast cancer risk. To confirm this interaction, we used the joint method and calculated the risk of breast cancer associated with both rs12539530 and *ESR1 *SNPs using a set of dummy variables representing different combinations of genotypes of *ESR1 *and rs12539530. In contrast to the increased, but not significant, risk associated with either a high-risk genotype of *ESR1 *or rs12539530 alone, the greatest risk was found in those patients harboring high-risk genotypes of both rs12539530 and *ESR1 *SNPs in all women combined (Figure 3). More interestingly, estrogen promotes breast tumorigenesis by forming a complex with the ER which then binds to EREs [1,2], and thus the relationship between breast cancer risk and the joint effect of rs12539530 and *ESR1 *polymorphism might be modified by estrogen exposure, which is consistent with what we observed (Figure 3). The risk of breast cancer associated with the joint effect of polymorphisms of rs12539530 and *ESR1 *was evaluated in different groups of women stratified by years of total estrogen exposure and a significant and increased joint effect was seen only in the subgroup of women with more than 30 years of estrogen exposure (Figure 3). In contrast, in the subset of women with less than 30 years of estrogen exposure, the same joint effect was associated with a nonsignificant aOR (Figure 3).

## Discussion

Of the predicted ERE-related sequences found throughout the whole genome, the present study has identified a genetic variation of rs12539530, a SNP located in the putative ERE site in intron 2 of *NRCAM*, as an important factor in determining susceptibility to breast cancer development. We attempted to address the possibility of false-positives and the effects of multiple testing by demonstrating a borderline significant *P *value in the permutation test. Even so, given the lack of strongly significant results, the power of the present study should be an issue of particular concern, and our suggestion that rs12539530 is involved in determining breast cancer susceptibility certainly needs to be confirmed in other studies with larger sample sizes.

In considering whether our findings represent a true association between this SNP in ERE and breast cancer, the most important issue is the interpretation of the identified association between SNPs and the trait. Because the SNP identified is in an intron, it does not affect amino acid coding and therefore probably does not directly affect protein function. In addition, the observed association between breast cancer and this SNP could be due to the presence of linkage disequilibrium (LD) between this SNP and other SNPs in exons (resulting in functional change) or in regulatory regions (affecting the expression of these genes). However, the first of these two possibilities is less likely, as we checked the LD block in which rs12539530 is located in the Chinese population in HapMap [24], which spans 22 kb in chromosome region 7q31, and found that the whole block is within intron 2 of *NRCAM*. In other words, no well-defined genes are in the same haplotype block as this SNP. To address the second possibility, we examined the sequence of this LD block and found that it contains more than 200 predicted transcription factor binding sites, including those for specificity protein 1 (Sp1) and activator protein 1 (AP-1). After blasting these binding sites with the sequences of 30 SNPs known to reside in this block as reported by HapMap, some SNPs were found to be located within these binding sites, so the second possibility cannot be totally excluded. However, we are prompted to suggest the breast tumorigenic contribution of rs12539530 on the basis of the findings that (1) rs12539530 and *ESR1 *SNPs jointly increased breast cancer susceptibility and (2) this joint effect was more significant in women with a longer period of estrogen exposure. In addition, this suggestion is mechanistically plausible and is similar to the finding that rs10736303, the SNP generating a putative ERE in intron 2 of *FGFR2*, has been identified as the most significant gene determining breast cancer susceptibility in recent genome-wide association studies (GWASs) [29-31]. In addition, expression of *NRCAM*, the gene putatively regulated by rs12539530, has been suggested to be upregulated in ER-positive, but not in ER-negative, breast cancer cell lines [25,26], and this hypothesis was confirmed by the present study (Figure 2).

Recent GWASs based on technological advances in high-throughput genotyping have provided information regarding the LD of neighboring polymorphisms. These GWASs have made possible the use of a few hundred thousand SNPs as tags for all other variants, as well as the biobanking of tens of thousands of specimens, allowing their immediate use in whole genome research. These studies have led to the mapping of novel susceptibility loci for many common traits, including breast cancer [30,31]. Interestingly, none of these loci identified as significant in determining breast cancer risk in GWASs have been identified as significant in previous association studies based on a candidate gene approach, and very few of the genes involved in the most plausible mechanisms of breast tumorigenesis, including those involved in DNA repair and sex hormone synthesis and metabolism pathways, have been reported as important in GWASs. Region 7q31, in which *NRCAM *is located, has never been reported to be important with regard to breast cancer risk determination in GWASs. This might be partially explained by the relatively low-penetrance effect of SNPs of estrogen-regulating genes. Distinct from those alleles identified by current GWAS, these polymorphic alleles of estrogen-regulating genes would predispose carriers to only a moderately increased risk of developing cancer. Thus, the significance of such SNPs depends not only on their own effect but also on the interaction between the target genes and other functionally related genes (for example, *ESR1*) or the promoting effect of reproductive risk factors reflecting estrogen exposure. Our findings are in line with this suggestion.

This study is a hybrid of candidate gene and genome-wide approaches in which we took advantage of both designs. The well-defined roles of the ER during breast tumorigenesis makes it reasonable to assume that polymorphic genetic variants of EREs, central nodes in the ER pathway, might underlie the variations seen between individuals in their susceptibility to breast cancer. This candidate mechanism lends critical support to the biological plausibility and tumorigenic relevance of our findings. In addition, our genotyping of SNPs on the basis of genome-wide predicted EREs provided a unique opportunity to comprehensively examine putative ERE sites without depending on a prior hypothesis. The successful identification of rs12539530 by using these combined methods suggests that this is a promising approach to identifying the breast tumorigenic contribution of EREs on a genome-wide scale on the basis of information generated by recent technological advances. For example, in our ongoing study, we are exploring the breast tumorigenic role of putative EREs identified using chromatin immunoprecipitation-based methods [25,32,33]. The simultaneous consideration of these ER-associated EREs and ER-regulating genes that drive breast tumorigenesis might be critical in the development of new anticancer drug targets and new therapeutic and diagnostic approaches.

## Conclusions

Though the promise of personalized medicine, in which the risk and the course of diseases and the efficacy of treatment protocols would be predicted on the basis of a person's genotype, must been tempered with caution, validated molecular tests assessing the patient's germline DNA already drive therapeutic decision-making. On the basis of the well-documented role of ER in breast cancer development and progression, this study explored whether genetic variations in EREs, the sequences bound by ER to activate the transcriptional regulation of target genes, are associated with susceptibility for breast cancer. Notably, the ERE sites genotyped were based on genome-wide prediction, providing a unique opportunity to comprehensively examine putative ERE sites without depending on a prior hypothesis. A significant combined effect of rs12539530, an ERE SNP in intron 2 of *NRCAM*, which codes for a cell adhesion molecule, and SNPs of *ESR1*, the gene coding for ER, on breast cancer risk was found. Our findings provide support for a role of *ESR1*-ERE polymorphism in determining susceptibility of breast cancer development. This knowledge will be helpful for directing the focus of future experimental studies.

## Abbreviations

CI: confidence interval; ER: estrogen receptor; ERα: estrogen receptor α; ERE: estrogen response element; FFTP: first full-term pregnancy; FTP: full-term pregnancy; GWAS: genome-wide association studies; LD: linkage disequilibrium; OR: odds ratio; SNP: single nucleotide polymorphism.

## Acknowledgements

Financial support was provided by grant NSC 98-2628-B-016-001-MY3 (to JCY) from the National Science Council, Taiwan and by grants TSGH-C97-7-S03, DOD-98-28-03 and DOD-98-36 from the Tri-Service General Hospital (to JCY and HMH).

## Competing interests

The authors declare that they have no competing interests.

## Authors' contributions

JCY and CYS participated in the study design and coordination, carried out the participant recruitment and drafted the manuscript. CNH and PEW carried out the genotyping and data analysis. BYB contributed to searching ERE websites using PReMod. HMH, STC and GCH carried out the participant recruitment. WCC and LYH contributed to the validation of *NRCAM *expression in breast cancer cell lines. SD and CWC performed *ESR1 *genotyping.

## Supplementary Material

Additional file 1 **Tables S1 and S2**. Table S1 presents genotype frequencies of sequence variants of estrogen response element (ERE)-related sequences in breast cancer patients and controls and the adjusted odds ratio (aOR) in relation to breast cancer risk. Table S2 presents breast cancer risk associated with genotypic polymorphism of rs12539530, a single-nucleotide polymorphism (SNP) in the estrogen response element (ERE)-related sequence, stratified by genotypes of *ESR1*, the estrogen receptor gene. Click here for file
