# Supplementary material for: Genetic variation in the genome-wide predicted estrogen response element-related sequences is associated with breast cancer development
Source: Breast Cancer Res. 2011 Jan 31;13(1):R13. doi: 10.1186/bcr2821 (PMC3109581; doi:10.1186/bcr2821)
Supplement: Additional file 1 — Tables S1 and S2. Table S1 presents genotype frequencies of sequence variants of estrogen response element (ERE)-related sequences in breast cancer patients and controls and the adjusted odds ratio (aOR) in relation to breast cancer risk. Table S2 presents breast cancer risk associated with genotypic polymorphism of rs12539530, a single-nucleotide polymorphism (SNP) in the estrogen response element (ERE)-related sequence, stratified by genotypes of ESR1, the estrogen receptor gene. [file bcr2821-S1.DOC]

Table S1. Genotype frequencies of sequence variants of estrogen-response- element (ERE)-related sequences in breast cancer cases and controls and the adjusted odds ratio (aOR) in relation to breast cancer risk

| SNP and  genotype* | | | No.  cases(%) | | | | No. controls(%) | | | | aOR  (95%CI)^†^ | | aOR for  trend^††^ | P for  permutation |
| --- | --- | --- | --- | --- | --- | --- | --- | --- | --- | --- | --- | --- | --- | --- |
| rs16930333 | | |  | | |  |  | | |  |  |  |  |  |
|  | | TT | 412 | | (51) |  | 420 | | (51) |  |  | 1.00(ref.)^**^ |  |  |
|  | | TA | 352 | | (43) |  | 334 | | (41) |  |  | 1.08(0.89-1.35) | 0.93 | 0.10 |
|  | | AA | 49 | | (6) |  | 66 | | (8) |  |  | 1.08(0.89-1.35) | (0.78-1.10) |  |
| rs7690530 | | |  | | |  |  | | |  |  |  |  |  |
|  | | AA | 229 | | (28) |  | 213 | | (26) |  |  | 1.00(ref.)^**^ |  |  |
|  | | AG | 392 | | (48) |  | 396 | | (48) |  |  | 0.95(0.74-1.22) | 0.93 | 0.34 |
|  | | GG | 193 | | (24) |  | 209 | | (26) |  |  | 0.87(0.65-1.17) | (0.81-1.08) |  |
| rs1378033 | | |  | | |  |  | | |  |  |  |  |  |
|  | CC | | 537 | (66) | |  | 543 | (66) | |  |  | 1.00(ref.)^**^ |  |  |
|  | CA | | 243 | (30) | |  | 244 | (30) | |  |  | 0.97(0.77-1.23) | 0.98 | 0.80 |
|  | AA | | 34 | (4) | |  | 32 | (4) | |  |  | 0.98(0.56-1.70) | (0.81-1.18) |  |
| rs2275675 | | |  | | |  |  | | |  |  |  |  |  |
|  | GG | | 257 | (32) | |  | 219 | (27) | |  |  | 1.00(ref.)^**^ |  |  |
|  | GA | | 381 | (47) | |  | 414 | (51) | |  |  | 0.84(0.66-1.08) | 0.93 | 1.00 |
|  | AA | | 174 | (21) | |  | 173 | (22) | |  |  | 0.87(0.65-1.18) | (0.80-1.08) |  |
| rs2249336 | | |  | | |  |  | | |  |  |  |  |  |
|  | TT | | 366 | (45) | |  | 366 | (46) | |  |  | 1.00(ref.)^**^ |  |  |
|  | TC | | 360 | (44) | |  | 356 | (43) | |  |  | 1.05(0.84-1.32) | 0.99 | 0.69 |
|  | CC | | 87 | (11) | |  | 94 | (11) | |  |  | 0.95(0.67-1.35) | (0.86-1.18) |  |
| rs12670356 | | |  | | |  |  | | |  |  |  |  |  |
|  | TT | | 238 | (29) | |  | 395 | (48) | |  |  | 1.00(ref.)^**^ |  |  |
|  | TC | | 296 | (49) | |  | 259 | (32) | |  |  | 1.04(0.81-1.33) | 1.03 | 0.43 |
|  | CC | | 178 | (22) | |  | 166 | (20) | |  |  | 1.06(0.80-1.42) | (0.89-1.19) |  |
| rs12868139 | | |  | | |  |  | | |  |  |  |  |  |
|  | CC | | 629 | (77) | |  | 618 | (75) | |  |  | 1.00(ref.)^**^ |  |  |
|  | CT | | 173 | (21) | |  | 186 | (23) | |  |  | 0.91(0.71-1.18) | 0.92 | 0.84 |
|  | TT | | 12 | (2) | |  | 14 | (2) | |  |  | 0.85(0.35-2.02) | (0.73-1.15) |  |
| rs4132275 | | |  | | |  |  | | |  |  |  |  |  |
|  | AA | | 291 | (36) | |  | 306 | (38) | |  |  | 1.00(ref.)^**^ |  |  |
|  | AG | | 388 | (48) | |  | 369 | (45) | |  |  | 1.16(0.92-1.47) | 1.03 | 0.51 |
|  | GG | | 134 | (16) | |  | 146 | (18) | |  |  | 1.00(0.74-1.37) | (0.89-1.19) |  |
| rs927855 | | |  | | |  |  | | |  |  |  |  |  |
|  | AA | | 671 | (82) | |  | 690 | (84) | |  |  | 1.00(ref.)^**^ |  |  |
|  | AC | | 134 | (17) | |  | 123 | (15) | |  |  | 1.16(0.87-1.55) | 1.18 | 0.81 |
|  | CC | | 9 | (1) | |  | 8 | (1) | |  |  | 1.59(0.56-4.49) | (0.92-1.53) |  |
| rs3806930 | | |  | | |  |  | | |  |  |  |  |  |
|  | AA | | 492 | (60) | |  | 479 | (59) | |  |  | 1.00(ref.) ^**^ |  |  |
|  | AC | | 283 | (35) | |  | 295 | (36) | |  |  | 0.85(0.68-1.07) | 0.92 | 0.65 |
|  | CC | | 39 | (5) | |  | 44 | (5) | |  |  | 1.04(0.64-1.71) | (0.77-1.10) |  |
| rs4375637 | | |  | | |  |  | | |  |  |  |  |  |
|  | GG | | 538 | (66) | |  | 535 | (65) | |  |  | 1.00(ref.)^**^ |  |  |
|  | GC | | 248 | (31) | |  | 246 | (30) | |  |  | 1.01(0.81-1.28) | 0.89 | 0.25 |
|  | CC | | 27 | (3) | |  | 37 | (5) | |  |  | 0.56(0.31-0.98) | (0.74-1.09) |  |
| rs4845952 | | |  | | |  |  | | |  |  |  |  |  |
|  | GG | | 383 | (47) | |  | 396 | (48) | |  |  | 1.00(ref.) |  |  |
|  | GA | | 358 | (43) | |  | 334 | (41) | |  |  | 1.07(0.86-1.33) | 0.98 | 0.68 |
|  | AA | | 84 | (10) | |  | 82 | (11) | |  |  | 0.89(0.62-1.27) | (0.84-1.15) |  |
| rs9374202 | | |  | | |  |  | | |  |  |  |  |  |
|  | TT | | 397 | (49) | |  | 419 | (51) | |  |  | 1.00(ref.)^**^ |  |  |
|  | TC | | 334 | (41) | |  | 325 | (40) | |  |  | 1.15(0.92-1.44) | 1.09 | 0.55 |
|  | CC | | 83 | (10) | |  | 76 | (9) | |  |  | 1.11(0.77-1.59) | (0.93-1.27) |  |
| rs12685327 | | |  | | |  |  | | |  |  |  |  |  |
|  | GG | | 531 | (65) | |  | 526 | (64) | |  |  | 1.00(ref.) ^**^ |  |  |
|  | GA | | 253 | (31) | |  | 257 | (32) | |  |  | 0.90(0.71-1.14) | 0.89 | 0.61 |
|  | AA | | 29 | (4) | |  | 34 | (4) | |  |  | 0.76(0.43-1.33) | (0.74-1.08) |  |
| rs7784390 | | |  | | |  |  | | |  |  |  |  |  |
|  | CC | | 436 | (54) | |  | 471 | (57) | |  |  | 1.00(ref.)^**^ |  |  |
|  | CT | | 319 | (39) | |  | 292 | (36) | |  |  | 1.23(0.98-1.53) | 1.12 | 0.93 |
|  | TT | | 59 | (7) | |  | 58 | (7) | |  |  | 1.08(0.72-1.64) | (0.95-1.32) |  |
| rs1939988 | | |  | | |  |  | | |  |  |  |  |  |
|  | AA | | 406 | (50) | |  | 388 | (48) | |  |  | 1.00(ref.)^**^ |  |  |
|  | AG | | 329 | (41) | |  | 353 | (44) | |  |  | 0.85(0.68-1.06) | 0.92 | 0.54 |
|  | GG | | 77 | (9) | |  | 69 | (8) | |  |  | 0.93(0.63-1.35) | (0.77-1.07) |  |
| rs12539530 | | |  | | |  |  | | |  |  |  |  |  |
|  | AA | | 254 | (31) | |  | 278 | (34) | |  |  | 1.00(ref.)^**^ |  |  |
|  | AG | | 392 | (48) | |  | 398 | (48) | |  |  | 1.16(0.91-1.47) | 1.18 | 0.08 |
|  | GG | | 169 | (21) | |  | 145 | (18) | |  |  | 1.40(1.04-1.90) | (1.02-1.37) |  |
|  | | |  | | |  |  | | |  |  |  |  |  |
| rs7210148 | | |  | | |  |  | | |  |  |  |  |  |
|  | TT | | 307 | (38) | |  | 308 | (38) | |  |  | 1.00(ref.)^**^ |  |  |
|  | TA | | 386 | (47) | |  | 376 | (46) | |  |  | 1.22(0.89-1.67) | 1.08 | 1.00 |
|  | AA | | 119 | (15) | |  | 130 | (16) | |  |  | 1.21(0.87-1.67) | (0.92-1.25) |  |
| rs6131771 | | |  | | |  |  | | |  |  |  |  |  |
|  | CC | | 238 | (30) | |  | 237 | (29) | |  |  | 1.00(ref.)^**^ |  |  |
|  | CA | | 414 | (51) | |  | 392 | (48) | |  |  | 1.14(0.90-1.46) | 0.99 | 0.08 |
|  | AA | | 154 | (19) | |  | 184 | (23) | |  |  | 0.96(0.71-1.30) | (0.86-1.16) |  |
| rs9527676 | | |  | | |  |  | | |  |  |  |  |  |
|  | AA | | 348 | (43) | |  | 379 | (46) | |  |  | 1.00(ref.)^**^ |  |  |
|  | AG | | 363 | (45) | |  | 367 | (45) | |  |  | 1.09(0.87-1.36) | 1.18 | 0.03 |
|  | GG | | 96 | (12) | |  | 71 | (9) | |  |  | 1.50(1.05-2.16) | (1.00-1.38) |  |
| rs11861016 | | |  | | |  |  | | |  |  |  |  |  |
|  | CC | | 609 | (75) | |  | 624 | (77) | |  |  | 1.00(ref.)^**^ |  |  |
|  | CT | | 190 | (24) | |  | 178 | (22) | |  |  | 1.14(0.88-1.46) | 1.07 | 0.83 |
|  | TT | | 10 | (1) | |  | 12 | (1) | |  |  | 0.74(0.28-1.93) | (0.86-1.35) |  |

*The “rs” number is the National Center for Biotechnology Information (NCBI) dbSNP cluster ID for each SNP.

^†^Adjusted odds ratio (aOR) and corresponding 95% confidence interval (95%CI) were calculated by logistic regression, in which we used a set of dummy variables representing different genotypes of SNPs of estrogen response elements. The age of the study participant, a family history of breast cancer, and reproductive risk factors were included in the regression model.

^††^The aOR for trend indicates the adjusted odds ratio associated with harboring one additional variant allele of individual SNPs, and it was estimated in the logistic regression model, in which the genotype was treated as a continuous variable.

^**^ref., reference group.

Table S2. Breast cancer risk associated with genotypic polymorphism of rs12539530, an SNP in the estrogen response element-related sequence, stratified by genotypes of *ESR1*.

|  |  | No. cases(%) | | No. controls(%) | |  | aOR(95%CI)* |  | No. cases(%) | | No. controls (%) | |  | aOR(95%CI)* |
| --- | --- | --- | --- | --- | --- | --- | --- | --- | --- | --- | --- | --- | --- | --- |
|  |  |  | |  | |  | rs3778609 of *ESR1* | | | |  | |  |  |
|  |  |  | | CC | |  |  |  |  | | CT/TT | |  |  |
| rs12539530 |  |  | |  | |  |  |  |  | |  | |  |  |
| AA |  | 101 | (30.8) | 135 | (36.5) |  | 1.00(ref.)^†^ |  | 64 | (29.1) | 109 | (30.8) |  | 1.00(ref.)^†^ |
| AG/GG |  | 227 | (69.2) | 235 | (63.5) |  | 1.43(1.01-2.02) |  | 156 | (70.9) | 245 | (69.2) |  | 1.24(0.82-1.88) |
|  |  |  | |  | |  | rs12665044 of *ESR1* | | | |  | |  |  |
|  |  |  | | CC | |  |  |  |  | | CT/TT | |  |  |
| rs12539530 |  |  |  |  |  |  |  |  |  |  |  |  |  |  |
| AA |  | 97 | (30.4) | 134 | (37.2) |  | 1.00(ref.)^†^ |  | 68 | (29.4) | 111 | (30.3) |  | 1.00(ref.)^†^ |
| AG/GG |  | 222 | (69.6) | 226 | (62.8) |  | 1.49(1.04-2.12) |  | 163 | (70.6) | 255 | (69.7) |  | 1.19(0.80-1.77) |
|  |  |  |  |  |  |  | rs827421 of *ESR1* | | | |  |  |  |  |
|  |  |  |  | AG/GG | |  |  |  |  |  | AA | |  |  |
| rs12539530 |  |  |  |  |  |  |  |  |  |  |  |  |  |  |
| AA |  | 88 | (28.9) | 147 | (32.3) |  | 1.00(ref.)^†^ |  | 77 | (31.3) | 99 | (36.4) |  | 1.00(ref.)^†^ |
| AG/GG |  | 217 | (71.2) | 308 | (67.7) |  | 1.40(1.00-1.98) |  | 169 | (68.7) | 173 | (63.6) |  | 1.19(0.78-1.8) |
|  |  |  |  |  |  |  | rs7739506 of *ESR1* | | | |  |  |  |  |
|  |  |  |  | AA | |  |  |  |  |  | AG/GG | |  |  |
| rs12539530 |  |  |  |  |  |  |  |  |  |  |  |  |  |  |
| AA |  | 130 | (28.5) | 191 | (34.1) |  | 1.00(ref.)^†^ |  | 34 | (38.6) | 50 | (33.6) |  | 1.00(ref.)^†^ |
| AG/GG |  | 327 | (71.5) | 369 | (65.9) |  | 1.43(1.06-1.92) |  | 54 | (64.1) | 99 | (66.4) |  | 0.82(0.43-1.55) |

*The adjusted odds ratio (aOR) and corresponding 95% confidence interval (95%CI) were estimated in a logistic regression model. The age of the study participant, a family history of breast cancer, and reproductive risk factors were included in the regression model.

^†^ref., reference group.
